# Digestibility and palatability of Virginia fanpetals (*Sida hermaphrodita* R.) silage in sheep

**DOI:** 10.5194/aab-65-89-2022

**Published:** 2022-02-22

**Authors:** Cezary Purwin, Marta Borsuk-Stanulewicz, Zenon Nogalski, Maja Baranowska, Aleksandra Zygmuntowicz, Jacek P. Michalski

**Affiliations:** 1 Department of Animal Nutrition and Feed Science, University of Warmia and Mazury in Olsztyn, Olsztyn, 10-719, Poland; 2 Department of Cattle Breeding and Milk Evaluation, University of Warmia and Mazury in Olsztyn, Olsztyn, 10-719, Poland; 3 Department of Pharmacology and Toxicology, University of Warmia and Mazury in Olsztyn, Olsztyn, 10-719, Poland; 4 Kielanowski Institute of Animal Physiology and Nutrition, Polish Academy of Sciences, Jabłonna, 05-110, Poland; ℹ previously published under the name Marta Borsuk

## Abstract

The aim of the current study is to evaluate Virginia fanpetals silage based on an apparent digestibility and palatability test performed on six adult rams. Alfalfa silage was used as standard forage for comparison. Virginia fanpetals samples were harvested in the bud-formation stage and alfalfa samples were harvested in the late bud stage. Virginia fanpetals silage had a crude protein (CP) content of 176 g kg ${}^{-1}$ dry matter (DM), a neutral detergent fiber (NDF) content of 378 g kg ${}^{-1}$ DM, and a lignin content of 42.8 g kg ${}^{-1}$ DM. Virginia fanpetals silage had higher acidity (pH of 4.30) and was characterized by intense lactic acid fermentation compared with alfalfa silage (80 % vs. 51 % of the total acids). The digestibility coefficient of Virginia fanpetals silage was as follows: for DM it was 0.707, for organic matter (OM) it was 0.724, for CP it was 0.861, and for NDF it was 0.609. In comparison with alfalfa silage, Virginia fanpetals silage was characterized by higher apparent digestibility of nutrients, but a significant difference was noted only for CP. The voluntary intake of Virginia fanpetals silage was significantly higher than that of alfalfa silage (1427.4 vs. 954 g DM). The greatest differences in voluntary intake were observed 0–2 and 8–12 h after feeding. Virginia fanpetals silage had a chemical composition similar to that of alfalfa, but it was characterized by a more desirable fermentation pattern and higher digestibility, and it was more willingly consumed by rams. The present findings indicate that Virginia fanpetals silage can be fed to sheep.

## 1 Introduction

In recent years, Virginia fanpetals (*Sida hermaphrodita* R.) has attracted the interest of European producers as a potential energy crop on account of its high yields (9–20 t ha ${}^{-1}$ of dry matter (DM) annually). Virginia fanpetals is a perennial plant native to North America that can be grown for 15–20 years. It has a complex root system that efficiently utilizes nutrients even in poor soils. The species regrows each year, increasing the number of shoots by 20–30 in successive years. At the end of the growing season, the branching stem of Virginia fanpetals exceeds 4 m in height, and it produces up to 40 shoots per square meter. The plant flowers from July until the first frost, which makes it a good source of nectar and pollen for honeybees. Virginia fanpetals can be used to improve soil stabilization, reduce the risk of soil erosion, increase the fertility and biological value of soils, and restore degraded soils. Virginia fanpetals biomass can be used in the pulp and paper industry and in the energy sector. Virginia fanpetals herbage has a high crude protein (CP) content of 308 to 139 g kg ${}^{-1}$ DM and a low lignin concentration of 64 to 99 g kg ${}^{-1}$ DM, depending on the growth stage (from 6 May to 25 June). First-cut biomass harvested in the bud-formation stage can be a valuable feed component, whereas second-cut biomass harvested at the end of the growing season can be used for energy production. The potential use of Virginia fanpetals as a fodder crop has also been investigated (Borkowska and Styk, 2006; Borkowska and Molas, 2012; Nahm and Morhart, 2018).

Previous studies have analyzed ruminal degradability and nutrient digestibility in sows and rabbits fed diets containing dehydrated Virginia fanpetals (Mroz and Tarkowski, 1991; Tarkowski and Truchliński, 2011; Purwin et al., 2019). It has also been found that Virginia fanpetals silage has a positive effect on carcass characteristics and meat quality in young bulls without compromising their fattening performance (Nogalski et al., 2020) and that it can be used as a partial substitute for alfalfa silage in dairy cow rations based on maize silage (Purwin et al., 2020). In both cited studies, the digestibility of diets containing Virginia fanpetals silage was determined, but the digestibility of the silage alone or its efficacy in sheep nutrition have not been investigated to date.

The aim of the current study is to evaluate the digestibility and palatability of Virginia fanpetals silage harvested in the bud-formation stage and fed to sheep.

## 2 Materials and methods

### 2.1 Silage

The experiment was conducted in 2015 in northeastern Poland (53 ${}^{\circ }$ 05 ${}^{\prime }$ 27.7 ${}^{\prime \prime }$ N, 21 ${}^{\circ }$ 11 ${}^{\prime }$ 47.5 ${}^{\prime \prime }$ E). At the beginning of the growing season, plants were fertilized with 100 kg nitrogen (N) per hectare, 50 kg potassium oxide (K ${}_{2}$ O) per hectare, and 80 kg phosphorus (V) oxide (P ${}_{2}$ O ${}_{5}$ ) per hectare. The experimental material was first-cut herbage of Virginia fanpetals harvested in the bud-formation stage (8 June) at a height of 25 cm with a Claas Jaguar 930 (GmbH, Harsewinkel, Germany) self-propelled forage harvester equipped with the Kemper 360 attachment. Alfalfa silage was made simultaneously. Alfalfa herbage was collected in a commercial plantation in the second year of its life cycle with a standard fertilization. The regrowth was harvested after 32 d at a height of 8 cm with the Claas Corto 270 mower-conditioner (GmbH, Harsewinkel, Germany); after 6 h of wilting, herbage was harvested with the same forage harvester. Herbage samples ( n=3 ) were collected before ensiling. Silage was ensiled in 220 L standard open-head high-density polyethylene (HDPE) drums ( n=3 ) (Brenntag GmbH, Essen, Germany) with drainage holes, and it was compressed to the density of 830 kg fresh matter (FM) per cubic meter. The silage was ensiled without additives. Virginia fanpetals silage was made from fresh herbage that could not be wilted due to unfavorable weather conditions. Alfalfa silage was made from wilted herbage, which contributed to its high quality. After 90 d, silage samples were collected with a probe ( ϕ 80 mm) along the entire length of the drums. A portion of the samples was dried at 60 ${}^{\circ }$ C or 48 h in the Binder FED 115 dryer (GmbH, Tuttlingen, Germany) and ground in the Retsch SK 100 mill (ZM 200, Retsch, Haan, Germany) to a 1 mm particle size. The remaining samples were frozen at -25 ${}^{\circ }$ C.

### 2.2 Chemical analysis of silage

Herbage and silage samples ( n=3 ) were assayed for proximate chemical composition, i.e., DM (method 934.01), CP (method 976.05), crude ash (method 942.05) as described by AOAC (2005), neutral detergent fiber (NDF) assayed with heat-stable amylase and expressed exclusive of residual ash, acid detergent fiber (ADF) expressed exclusive of residual ash, and acid detergent lignin (ADL) using the ANKOM220 fiber analyzer (ANKOM Technology Corp., Macedon, NY, USA) following Van Soest et al. (1991). Non-protein nitrogen (NPN) was calculated as a difference between total nitrogen (TN) and protein nitrogen determined with the use of trichloroacetic acid (TCA), as described by Licitria et al. (1996). The content of ammonia nitrogen (N-NH ${}_{3}$ ) was determined by direct distillation using the 2100 Kjeltec distillation unit (FOSS Analytical A/S, Hilleröd, Denmark) after increasing the pH of the samples by adding magnesium oxide (MgO); acidity was measured with the HI 8314 pH meter (Hanna Instruments, Woonsocket, RI, USA). The concentrations of acetic acid and butyric acid were determined by gas chromatography using a Varian 450-GC system coupled with a flame ionization detector (FID) and a 25 m long capillary column CP-FFAP (the internal diameter was 0.53 mm, and the thickness of the coating film was 1.0 µ m). Lactic acid was determined by high-performance liquid chromatography (HPLC Shimadzu) on a MetaCarb 67H P/N 5244 column (Varian, Palo Alto, CA, USA) with 0.0025 M sulfuric acid as the mobile phase, according to the manufacturer's protocol (Purwin et al., 2020). Silage quality was assessed according to the DLG Key (Weissbach and Honig, 1992). The chemical composition and fermentation products of Virginia fanpetals and alfalfa are presented in Table 1. The physical structure of the silage was also determined and is presented in Table 2.

**Table 1:** Chemical composition and fermentation products (g kg ${}^{-1}$ of DM) of silage.

Specification	Virginia fanpetals		Alfalfa	
	herbage	silage	herbage	silage
Dry matter (g kg ${}^{-1}$ )	224.000	199.000	188.000	266.000
Crude ash (g kg ${}^{-1}$ DM)	78.100	88.900	70.800	117.000
Crude protein (g kg ${}^{-1}$ DM)	182.000	176.000	185.000	197.000
Ether extract (g kg ${}^{-1}$ DM)	19.900	21.100	19.200	21.000
NDF (g kg ${}^{-1}$ DM)	375.000	378.000	462.000	466.000
ADF (g kg ${}^{-1}$ DM)	289.000	314.000	383.000	384.000
ADL (g kg ${}^{-1}$ DM)	33.300	42.800	86.800	72.500
ADL / NDF ratio	0.089	0.113	0.188	0.156
NFC (g kg ${}^{-1}$ DM)	345.000	336.000	263.00	199.000
NPN (g kg ${}^{-1}$ TN)	274.000	683.000	422.00	700.000
N-NH ${}_{3}$ (g kg ${}^{-1}$ TN)	n/a	100.000	n/a	42.000
pH	n/a	4.300	n/a	4.670
Lactic acid (g kg ${}^{-1}$ DM)	n/a	114.000	n/a	33.300
Acetic acid (g kg ${}^{-1}$ DM)	n/a	19.500	n/a	25.500
Butyric acid (g kg ${}^{-1}$ DM)	n/a	8.570	n/a	6.770
Silage quality according to the DLG Key				
Points	n/a	75.000	n/a	75.000
Quality	n/a	good	n/a	good

NDF stands for neutral detergent fiber. ADF stands for acid detergent fiber. ADL stands for acid detergent lignin. NFC stands for non-fiber carbohydrate; NFC was calculated according to the NRC (2001) standard according to the following equation: NFC = 1000 - Ash - CP - EE - NDF (NFC is the fraction of the dry matter of the feed minus crude ash, crude protein, extract ether and neutral detergent fiber). All concentrations are expressed as grams per kilogram of DM. NPN stands for non-protein nitrogen, TN stands for total nitrogen, N-NH ${}_{3}$ stands for ammonia nitrogen, and n/a stands for not applicable.

**Table 2:** Particle length (grams of DM per kilogram of DM).

Specification	Alfalfa	Virginia fanpetals	SEM
	silage	silage	
>19.05 mm	232.000	148.000	42.700
7.87–19.05 mm	408.000	418.000	23.400
1.78–7.87 mm	323.000	401.000	4.760
<1.78 mm	27.000	33.000	1.460

Particle size distribution was determined using the Penn State Particle Separator containing three sieves (19.05, 7.87, and 1.78 mm). SEM stands for standard error of mean.

### 2.3 Sheep-feeding trials

Silage digestibility and palatability were evaluated in the same six adult Polish Merino rams in the 2×3 design. The trial was carried out in accordance with EU Directive 2010/63/EU on the protection of animals used for scientific purposes (OJEU, 2010). The research did not require the approval of the local ethics committee.

### 2.4 Palatability test

A palatability test of Virginia fanpetals silage and alfalfa silage was performed on six adult Polish Merino rams (with an average body weight of 80 kg ± 3.74 kg). The animals were kept in individual pens measuring 0.8 m × 1.3 m with free access to water; openwork partitions were used so that the animals could keep eye contact. During a 7 d adjustment period, all rams were fed meadow hay ad libitum. The palatability trial proper lasted for 5 d. The analyzed silage was offered once daily in the amount of 5 kg. The position of containers was changed each time during silage intake control. Feed leftovers were weighed 2, 4, 6, 8, 12, and 24 h after the first feeding. Leftovers were weighed each day, and feed and leftover samples were collected to precisely determine silage DM intake by the rams.

### 2.5 Silage digestibility

The apparent digestibility of DM, organic matter (OM), CP, and NDF was determined by the balance method in six adult Polish Merino rams (with average body weight of 80 kg) kept in individual pens with faecal collection bags. Silage was the only forage, and it was fed ad libitum twice daily (07:30 and 17:30 CET, GMT + 1). After a 14 d adjustment period, faeces and leftover feed were collected for 5 d; 10 % of faeces and leftover samples were collected and weighed twice daily. Leftovers were weighed, and bulk samples collected from each animal were averaged. The samples were frozen at -25 ${}^{\circ }$ C, and faeces samples from each animal were used (after thawing) to prepare a bulk sample that was homogenized. Analytical samples were collected for TN determination using the Kjeldahl method. The remaining faeces and leftover samples were dried at 60 ${}^{\circ }$ C for 72 h and ground to pass through a 1 mm screen. Faeces and leftover samples were assayed for the content of DM (method 934.01), crude ash (method 942.05), CP (method 976.05) (AOAC, 2005), and NDF (Van Soest et al., 1991).

### 2.6 Calculations and statistical analyses

Dry matter content was adjusted for drying at 60 ${}^{\circ }$ C with the use of the equation proposed by Porter and Murray (2001). The effect of the ensiling process on the apparent digestibility and palatability of Virginia fanpetals silage was analyzed. The results were presented as means and standard errors of the mean (SEM). The data were processed statistically by an analysis of variance method (one-way ANOVA). The significance of differences between mean values was determined by. The results were analyzed statistically using STATISTICA v. 12.0 software (2014).

## 3 Results

### 3.1 Palatability test

**Figure 1:**
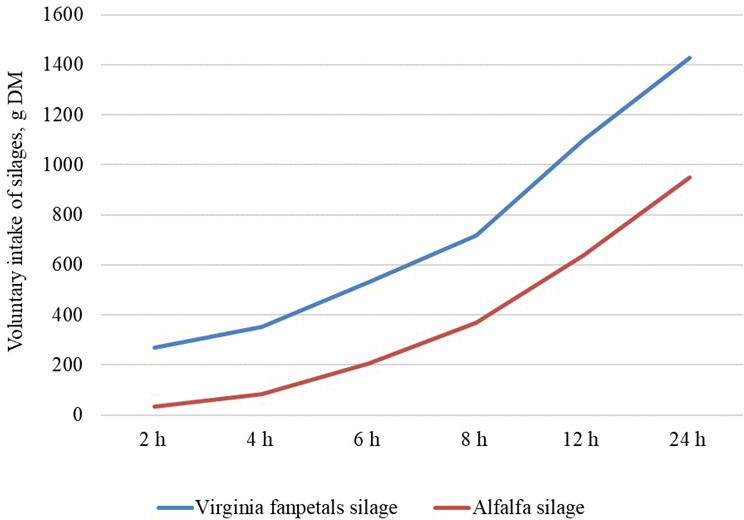
Rate of voluntary intake of silage in a preference test (g DM).

The daily voluntary intake of Virginia fanpetals silage was 1.5 times that of alfalfa silage ( P<0.010 ) (Table 3), which points to the higher palatability of the former (Fig. 1). Throughout the palatability trial, Virginia fanpetals silage was consumed in larger quantities than alfalfa silage, and the greatest differences in silage intake were observed 0–2 h ( P<0.010 ) and 8–12 h ( P=0.046 ) after feeding. Silage intake after 12 h was highly similar in both groups ( P=0.657 ).

**Table 3:** Voluntary intake of silage in a preference test (g DM).

Specification	Virginia fanpetals	Alfalfa	SEM	p value
	silage	silage		
0–2 h	270.000	34.800	37.400	< 0.010
2–4 h	83.400	48.200	10.100	0.079
4–6 h	177.000	128.000	15.300	0.113
6–8 h	187.000	163.000	18.500	0.529
8–12 h	382.000	269.000	19.100	0.046
12–24 h	328.000	311.000	17.400	0.657
Total	1427.400	954.000	74.900	< 0.010

SEM stands for standard error of the mean.

### 3.2 Apparent digestibility

Virginia fanpetals silage was characterized by numerically higher apparent digestibility of all analyzed nutrients compared with alfalfa silage used as standard forage, but a significant ( P<0.001 ) difference was noted only for CP digestibility (Table 4). An analysis of the chemical composition (Table 1) and nutrient digestibility of both silage types revealed that Virginia fanpetals silage was characterized by a higher content of only one digestible nutrient, i.e., CP (151.5 g kg ${}^{-1}$ DM), when compared with alfalfa silage (135.7 g kg ${}^{-1}$ DM).

**Table 4:** Apparent digestibility and D value (g kg ${}^{-1}$ DM) of silage.

Specification	DM	OM	CP	NDF	D value
Virginia fanpetals silage	0.707	0.724	0.861	0.609	660.000
Alfalfa silage	0.679	0.707	0.689	0.588	624.000
SEM	0.007	0.006	0.024	0.008	7.000
P value	0.109	0.169	<0.010	0.282	0.005

DM stands for dry matter. OM stands for organic matter. CP stands for crude protein. NDF stands for neutral detergent fiber. D -value is the amount of digestible organic matter in dry matter. SEM is the standard error of the mean.

## 4 Discussion

### 4.1 Palatability test

Silage intake is affected by the digestibility and content of cell walls (Dawson et al., 1999; Wright et al., 2000), the content of fermentation products, and modifications of carbohydrate and nitrogen fractions during fermentation (Huhtanen et al., 2002). In comparison with alfalfa silage, Virginia fanpetals silage was characterized by a lower content of DM and NDF (Table 1). The total voluntary intake of both silage types on a DM basis was high (Table 3) compared with the silage intake by finishing lambs (mean live weight 29.4 ± 0.66 kg) (diets supplemented with molassed sugar beet pellets) reported by Speijers et al. (2005): alfalfa silage had a value of 660 g DM, red clover silage had a value of 800 g DM, and ryegrass silage had a value of 580 g DM. In the present study, rams more willingly consumed silage with a lower DM content (Virginia fanpetals silage; see Table 1). The higher intake of Virginia fanpetals silage could result from its lower NDF content and the composition of cell walls. In a study by Van Soest (1994), the coefficients of correlation between NDF content and the ADL / NDF ratio vs. cell wall digestibility were -0.81 and -0.90 , respectively.

In comparison with alfalfa silage, Virginia fanpetals silage was characterized by a more desirable composition of NDF, a more desirable ratio of lactic acid to acetic acid, better acidity, and a similar concentration of butyric acid (Table 1). According to the DLG Key, the quality of both silage types was good, but both silage types had a high concentration of butyric acid (Table 1). In the group of fermentation products, ammonia has a direct negative effect on the taste and smell of silage, and ammonia concentration is positively correlated with the content of other protein degradation products affecting the taste of silage and the hemostatic regulation of silage intake (Huhtanen et al., 2002). In the two silage types, the content of N-NH ${}_{3}$ was 100 (fanpetals) and 42 g kg ${}^{-1}$ TN (alfalfa), respectively, and the minimal difference indicates that it had no influence on silage intake or the feed preferences of rams. According to Rook and Gill (1990), ammonia has a low direct impact on silage intake, but its content is related with the content of other fermentation products, such as volatile fatty acids and other nitrogen compounds. The above can explain the low coefficient of correlation between ammonia content and silage intake when ammonia concentration is expressed in terms of DM content and not TN content. Research shows that the only product that has an adverse effect on silage palatability is acetic acid, either alone (Baumont, 1996) or in combination with low pH and high concentrations of other acids (Buchanan-Smith, 1990). In the present study, rams preferred silage with a lower content of acetic acid, which accounted for 14 % and 39 % of total acids in Virginia fanpetals silage and alfalfa silage, respectively. Meeske et al. (1999) demonstrated that the concentration of lactic acid up to 100 g kg ${}^{-1}$ DM was positively correlated with silage intake, whereas Thomas et al. (1980) found that an increase in lactic acid content from 135 to 180 g kg ${}^{-1}$ DM decreased silage intake. Lactic acid concentration and pH point to a desirable fermentation pattern (McDonald, 1991); it appears that a large part of N-NH ${}_{3}$ in Virginia fanpetals silage did not come from protein degradation, as confirmed by the high NPN content of herbage (Table 1). In the current study, rams also preferred silage with higher lactic acid content (114 g kg ${}^{-1}$ DM). The negative correlation between butyric acid concentration and silage intake observed by Rook and Gill (1990) was not confirmed in our study. According to Miettinen et al. (1991), silage intake was reduced by 30 % when the total content of organic acids exceeded 130 g kg ${}^{-1}$ DM. Such an observation was not made in our study, where rams preferred Virginia fanpetals silage to alfalfa silage, although the former had a 2-fold higher total acid content (142 g kg ${}^{-1}$ DM). The intake of Virginia fanpetals silage would be higher if a fermentation inhibitor were used (Huhtanen et al., 2002).

In the current experiment, the moisture content of Virginia fanpetals silage and alfalfa silage had no direct negative influence on DM intake. This indicates that high moisture content decreases silage intake because it is associated with higher concentrations of fermentation products that adversely affect intake, i.e. acetic acid, butyric acid, and ammonia (Dulphy and Van Os, 1996; Manyawu et al., 2003). In the present study, an analysis of silage intake in the diurnal cycle revealed the greatest differences between the silage types within 0–2 h after feeding. In this time interval, the levels of metabolites in silage had the most significant effect on intake. Chiofalo et al. (1992) reported that the lower intake of the less palatable silage resulted mostly from the fact that smaller amounts of silage were consumed within the first few hours after feeding. The results of the current study indicate that Virginia fanpetals silage can be willingly consumed by ruminants. Adult rams consumed larger amounts of Virginia fanpetals silage, and throughout the experiment they preferred silage with higher moisture content; higher concentrations of lactic acid, butyric acid, and ammonia; and lower pH.

### 4.2 Apparent digestibility

The apparent digestibility of the control alfalfa silage was higher than the values reported by Nadeau et al. (2000) in a study of lambs wherein the digestibility coefficient of DM was 0.619 and NDF was 0.409. In the cited study, the concentrations of NDF (432 g kg ${}^{-1}$ DM) and ADL (73 g kg ${}^{-1}$ DM) were comparable with those noted in alfalfa silage in the present experiment (466 and 72.5 g kg ${}^{-1}$ DM, respectively). Differences in nutrient digestibility between experimental animals can result from age-related changes in digestive function (Cruickshank et al., 1990). In a study by Tarkowski (2006), dehydrated Virginia fanpetals with a CP content of 185 g kg ${}^{-1}$ DM fed to sheep had a higher DM digestibility coefficient (0.741) and lower CP digestibility coefficient (0.701).

The higher DM digestibility coefficient of Virginia fanpetals silage compared to alfalfa silage could be due to the higher digestibility of OM, resulting from the higher concentration of non-fiber carbohydrates (NFC) (Table 1), and higher digestibility of CP and NDF (Table 4). Since the concentrations of readily digestible hemicellulose in NDF were similar in both silage types (0.169 in Virginia fanpetals silage vs. 0.175 in alfalfa silage), the higher digestibility of cell walls in Virginia fanpetals silage could result from a lower degree of lignification (Noziere et al., 2010), as confirmed by the ADL / NDF ratio (0.113 in Virginia fanpetals silage and 0.156 in alfalfa silage).

Nutrient digestion involves the breakdown of feed into smaller particles and microbial colonization, and the processes of swallowing and chewing promote saliva production, enzyme secretion, and hydrolysis (Sauvant et al., 1990). The higher digestibility of Virginia fanpetals silage could result from differences in NDF concentration and, as a consequence, differences in the ruminal retention time of feed particles (Noziere et al., 2010), which affects digestibility. The retention time of legume particles was found to be shorter than that of grass particles (Dewhurst et al., 2003). The differences in the digestibility of both silage types could also be due to their different physical structure, although herbage was harvested with the same forage harvester. Unlike alfalfa herbage, Virginia fanpetals herbage was not simply chopped into small pieces. Its stems were separated into two fractions: a fraction of small particles of crushed cortex and a fraction of soft cellulose fibers that had not been cut and were thus much longer than 10 mm.

The higher digestibility of CP in Virginia fanpetals silage may be due to the larger amount of protein supplied to the small intestine of ruminants, determined by the greater extent of microbial protein synthesis, whose efficiency is affected by rumen-available energy derived mostly from carbohydrate fermentation (Hvelplund and Weisbjerg, 2000). In the current study, Virginia fanpetals silage had higher NFC content (336 g kg ${}^{-1}$ DM) than alfalfa silage (199 g kg ${}^{-1}$ DM).

## 5 Conclusions

Virginia fanpetals can be used not only as a source of renewable energy but also as supplementary forage for ruminants. The palatability test revealed that Virginia fanpetals silage can be willingly consumed by animals provided that the fermentation pattern is adequate. In comparison with alfalfa silage, Virginia fanpetals silage had similar protein content but a lower content of NDF with a more desirable composition and higher digestibility. As a result, adult rams preferred Virginia fanpetals silage despite its higher moisture content and a less desirable fermentation pattern. The results of the current study indicate that Virginia fanpetals silage can be fed to adult sheep. Further research is also needed to develop the optimal production technology for Virginia fanpetals silage.

## Data Availability

The datasets generated and/or analyzed during the current study are available from the corresponding author on reasonable request.
